# Supplementary material for: Estimating the Postmortem Interval of Wild Boar Carcasses
Source: Vet Sci. 2020 Jan 5;7(1):6. doi: 10.3390/vetsci7010006 (PMC7157510; doi:10.3390/vetsci7010006)
Supplement: Supplementary file 1 [file vetsci-07-00006-s001.zip › Supplementary Table S2.docx]

**Table S2.** Partial body scoring (PBS) scheme of Keough et al. (2017), compared to the PBS scheme adapted to wild boar as used in the present study. The numbers indicate the assigned scores for each change (in brackets the scores assigned by Keough et al. 2017).

| **Stage** | **Keough et al. (2017)** | **Present study** | **Score** |
| --- | --- | --- | --- |
| **Head - fresh** | Fresh, no discoloration – slight lividity (pink/red) |  | (1) |
| **Head - early decomposition** | Insect activity; pronounced lividity (dark pink/red) |  | (2) |
|  | Dark-red discoloration with some flesh still relatively fresh; oedema of ears; maggot colonization (mouth); initial bloating of neck and skin slippage |  | (3) |
|  | Discoloration and/or brownish shades particularly at edges, drying of nose, ears, and lips; prominent bloating of neck; maggot colonization (mouth and eyes); purging of decompositional fluids (mouth) |  | (4) |
|  | Purging of decompositional fluids (mouth, eyes, nose); brown discoloration; hair loss and skin slippage; drying of lips, nose and ears | Purging of decompositional fluids from mouth and nose | (5) 1 |
|  | Black discoloration of flesh; extensive maggot colonization and migration |  | (6) |
| **Head - advanced decomposition** | Caving in of the flesh and tissues of eyes and throat | Moist decomposition | (7) 2 |
|  | Moist decomposition with bone exposure less than one half that of the area being scored |  | (8) |
|  | Mummification with bone exposure less than one half that of the area being scored | First signs of bone exposure | (9) 3 |
| **Head - skeletonization** | Bone exposure of more than half of the area being scored with greasy substances and decomposed tissue | Some bones exposed, but still covered with tissue | (10) 4 |
|  | Bone exposure of more than half the area being scored with desiccation of mummified tissue |  | (11) |
|  | Bones largely dry, but retaining some grease | Bones only covered with desiccated skin; no soft tissues left | (12) 5 |
|  | Dry bone |  | (13) |
| **Trunk - fresh** | Fresh, no discoloration – slight lividity (pink) | Algor mortis; no visible swelling; typical wild boar (no putrefaction) odor | (1) 1 |
| **Trunk - early decomposition** | Skin appears shiny/glossy with early bloating and may show purple-black discoloration over abdominal area | Discoloration of the skin (blue-green) | (2) 2 |
|  | Gray-purple to green discoloration: some flesh still relatively fresh; marbling of abdomen with maximum bloat | Bloating | (3) 3 |
|  | Purple-black discoloration and purging of decompositional fluids; skin slippage with maggot-filled blisters present; hair loss | Skin slippage or loss of bristles | (4) 4 |
|  | Post-bloating following release of the abdominal gases, with extensive skin slippage and drying out of blisters | Opening of the abdominal cavity; strong putrefaction odor | (5) 5 |
| **Trunk -advanced decomposition** | Decomposition of tissue producing sagging of flesh; caving in of the abdominal cavity | Liquefaction of tissues; Purging of decomposition fluids into the surrounding | (6) 6 |
|  | Moist decomposition with bone exposure less than one half that of the area being scored | Decrease in biomass;  Caving in of the abdomen | (7) 7 |
|  | Mummification with bone exposure less than one half that or the area being scored | Most soft tissues are gone; remaining substance is dry/ hard/ cheesy/ mummified or black and sticky;  Little putrefaction smell | (8) 8 |
| **Trunk - skeletonization** | Bones with decomposed tissue, sometimes with body fluids and grease still present | Bones easily palpable under the skin, but still covered with tissue | (9) 9 |
|  | Bones with desiccated or mummified tissue covering less than one half of the area being scored | Clean bones, desiccated skin and bunches of bristles left; Fungi, algae, moss or lichen grow on skin and bones | (10) 10 |
|  | Bones largely dry, but retaining some grease | Only disarticulated, bleached bones left | (11) 11 |
|  | Dry bone |  | (12) |
| **Limbs - fresh** | Fresh, no discoloration – slight lividity (pink) with rigor present | Rigor mortis | (1) 1 |
| **Limbs - early decomposition** | Pink-white appearance with bloating of proximal parts of limbs |  | (2) |
|  | Gray to green discoloration; marbling and shiny appearance of skin; some flesh still relatively fresh; skin slippage and hair loss |  | (3) |
|  | Discoloration and/or brownish shades particularly at edges, drying of skin (starting distal and proximal) |  | (4) |
|  | Brown to black discoloration, skin having a leathery appearance |  | (5) |
| **Limbs - advanced decomposition** | Moist decomposition with bone exposure less than one half that of the area being scored | Loss of soft tissues | (6) 2 |
|  | Mummification with bone exposure of less than half of the area being scored |  | (7) |
| **Limbs -skeletonization** | Bone exposure over one half of the area being scored, some decomposed tissue and body fluids remaining | Some bones exposed, but still covered with tissue | (8) 3 |
|  | Bones largely dry, but retaining some grease |  | (9) |
|  | Dry bone | Bones only covered with desiccated skin; no soft tissues left | (10) 4 |
