# Supplementary figures and images for: Estimating the Postmortem Interval of Wild Boar Carcasses
Source: Vet Sci. 2020 Jan 5;7(1):6. doi: 10.3390/vetsci7010006 (PMC7157510; doi:10.3390/vetsci7010006)

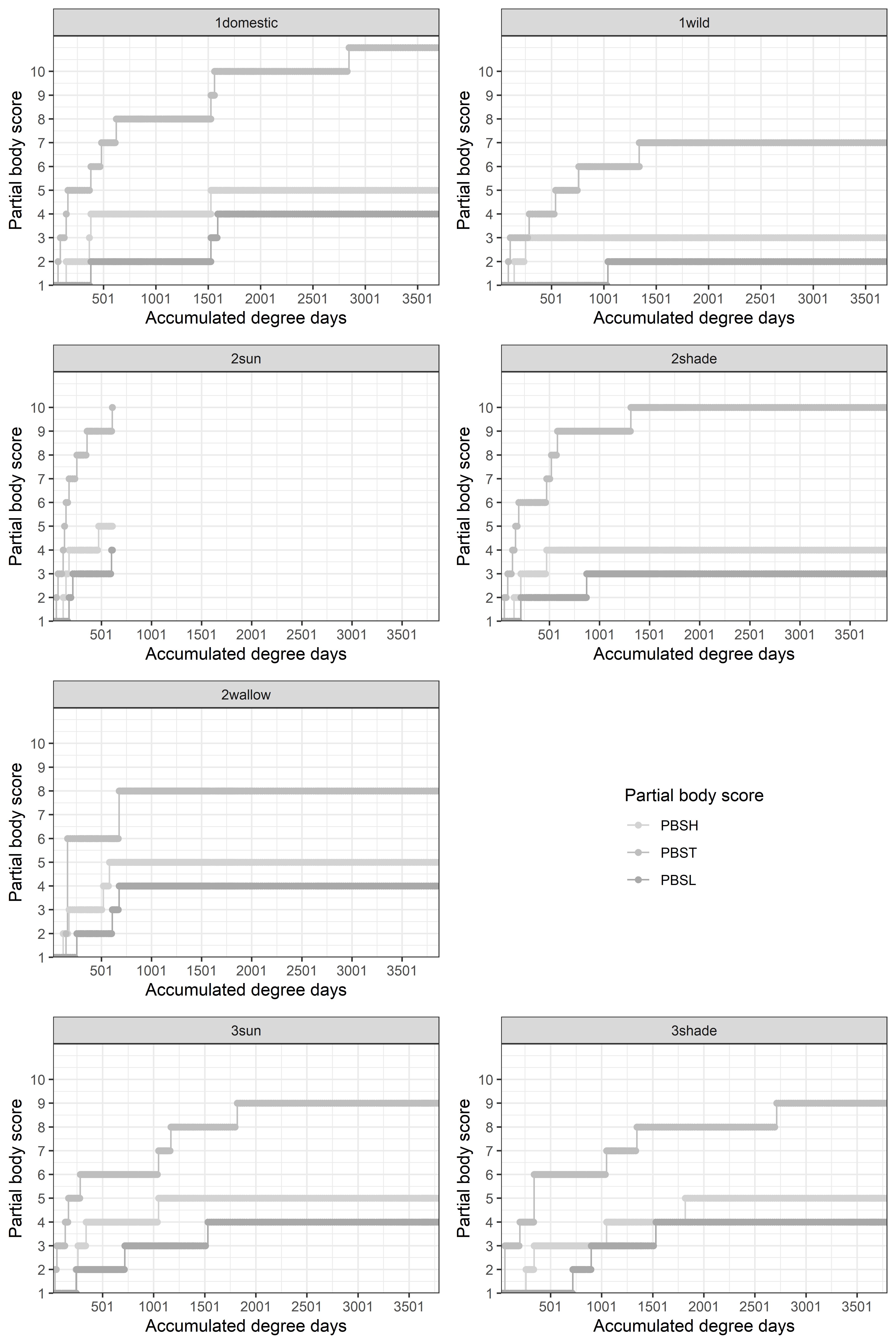

Supplement: Supplementary file 1 [file vetsci-07-00006-s001.zip › Supplementary Figure S1.jpeg]
